# *N*-methyl-2-pyridone-5-carboxamide (2PY)—Major Metabolite of Nicotinamide: An Update on an Old Uremic Toxin

**DOI:** 10.3390/toxins8110339

**Published:** 2016-11-15

**Authors:** Aurélie Lenglet, Sophie Liabeuf, Sandra Bodeau, Loïc Louvet, Aurélien Mary, Agnès Boullier, Anne Sophie Lemaire-Hurtel, Alexia Jonet, Pascal Sonnet, Said Kamel, Ziad A. Massy

**Affiliations:** 1 Institut National de la Santé et de la Recherche Médicale (INSERM U-1088), Jules Verne University of Picardie, Amiens 80000, France; terrier-lenglet.aurelie@chu-amiens.fr (A.L.); liabeuf.sophie@chu-amiens.fr (S.L.); bodeau.sandra@chu-amiens.fr (S.B.); loic.louvet@u-picardie.fr (L.L.); mary.aurelien@chu-amiens.fr (A.M.); agnes.boullier@u-picardie.fr (A.B.); said.kamel@u-picardie.fr (S.K.); 2 Department of Pharmacy, Amiens University Medical Center, Amiens 80000, France; 3 Clinical Research Centre and Division of Clinical Pharmacology, Amiens University Medical Center, Amiens 80000, France; 4 Laboratory of Pharmacology and Toxicology, Amiens University Medical Center, Amiens 80000, France; lemaire-hurtel.anne-sophie@chu-amiens.fr; 5 Biochemistry Laboratory, Amiens University Medical Center, Amiens 80000, France; 6 Laboratory of Glycochimie, des Antimicrobiens et des Agroressouces, Unité Mixte de Recherche—Centre National de la Recherché Scientifique (UMR CNRS) 7378, UFR de Pharmacy, Jules Verne University of Picardie, Amiens 80000, France; alexia.jonet@u-picardie.fr (A.J.); pascal.sonnet@u-picardie.fr (P.S.); 7 Division of Nephrology, Ambroise Paré University Medical Center, Assistance Publique—Hôpitaux de Paris APHP, Boulogne, Billancourt, Paris 92100, France; 8 INSERM U1018, Team 5, CESP (Centre de Recherche en Épidémiologie et Santé des Populations), Paris-Saclay University, and Paris Ouest-Versailles-Saint-Quentin-en-Yvelines University (UVSQ), Villejuif 94800, France

**Keywords:** *N*-methyl-2-pyridone-5-carboxamide, uremic toxin, nicotinamide, niacin, chronic kidney disease

## Abstract

*N*-methyl-2-pyridone-5-carboxamide (2PY, a major metabolite of nicotinamide, NAM) was recently identified as a uremic toxin. Recent interventional trials using NAM to treat high levels of phosphorus in end-stage renal disease have highlighted new potential uremic toxicities of 2PY. In the context of uremia, the accumulation of 2PY could be harmful—perhaps by inhibiting poly (ADP-ribose) polymerase-1 activity. Here, we review recently published data on 2PY’s metabolism and toxicological profile.

## 1. Introduction

Nicotinamide (NAM) is a water-soluble amine derivative of nicotinic acid (niacin, vitamin B3). The latter vitamin is essential for the body’s metabolism of carbohydrates, fats and many other substances. It has been shown that treatment with NAM decreases the phosphate load by inhibiting the sodium-phosphate co-transporters NaPi2a and NaPi2b (respectively located in the renal proximal tubule and the intestine) [1,2,3]. Many researchers have explored the potential of NAM and its analogs for phosphate control in end-stage renal disease (ESRD) [4]. *N*-methyl-2-pyridone-5-carboxamide (2PY) is a pyridine derivative of NAM and is one of NAM’s main metabolites in humans. The European Uremic Toxins (EUTox) working group [5] has classified 2PY as a uremic toxin and has included it in the group of low-molecular-weight, water-soluble, non-protein-bound molecules that can easily be removed by dialysis [6]. However, recent interventional trials in which NAM is used to treat high phosphorus levels in ESRD patients have brought to light new potential uremic toxicities associated with 2PY. In fact, circulating 2PY levels were found to be hugely elevated in NAM-supplemented ESRD patients, relative to non-supplemented ESRD patients [7]. We therefore decided to (i) review recently published data related to 2PY’s metabolism and role as a uremic toxin, and (ii) consider the potential toxic consequences of massive 2PY accumulation in the context of NAM supplementation.

## 2. Metabolism of NAM and Production of 2PY

The body can use dietary nicotinic acid (niacin, vitamin B3), NAM or tryptophan to synthesize nicotinamide adenine dinucleotide (NAD) as a substrate of the so-called NAD donor. Nicotinic acid (synthesized by plants and algae) is the basic precursor in the food chain, whereas NAM is a degradation product of the pyridine nucleotides; NAM thus constitutes the predominant form of vitamin B3 absorbed from animal-based foods [8]. It should be stressed that although NAM and niacin have similar functions as vitamins, their respective pharmacological and toxicological properties differ [9].

**Figure 1:**
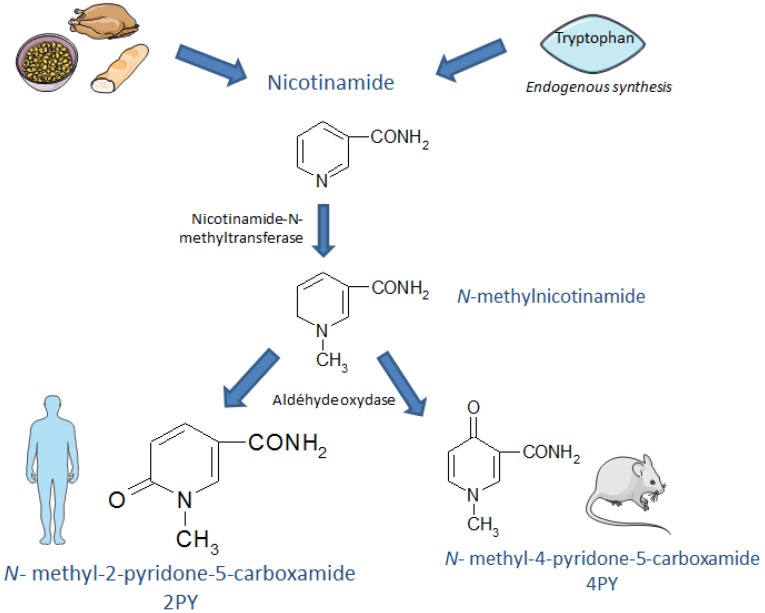
The metabolic production of *N*-methyl-2-pyridone-5-carboxamide.

NAM is metabolized in the liver by cytochrome P450 to variously form nicotinamide-*N*-oxide (via an oxidative reaction), 6-hydroxy-nicotinamide (via a hydroxylation reaction) and *N*-methyl-nicotinamide (MNA, in a reaction catalyzed by nicotinamide-*N*-methyltransferase). In mammals, MNA is further metabolized to 2PY or *N*-methyl-4-pyridone-5-carboxamide (4PY) by aldehyde oxidase. The 2PY/4PY ratio depends on the species and gender. NAM catabolism is schematically represented in Figure 1.

A number of analytical methods (such as high-performance liquid chromatography with ultraviolet or fluorescence detection, and liquid chromatography with tandem mass spectrometry (LC-MS/MS)) can be used to determine the concentration of 2PY [10,11,12,13,14]. Mullangi et al. have discussed the drawbacks and the advantages of the various methods reported [15].

NAM disappears rapidly from the circulation and distributes into all tissues. In humans, NAM is excreted in the urine as the *N*-methyl derivate. A pharmacokinetic study of healthy volunteers showed that after oral administration of niacin (2 g of extended released form), the urine recovery of niacin and metabolites accounted for 69.5% of the administered dose, and only 3.2% was excreted as unmodified niacin. The highest recovery was observed for 2PY (37.9%), followed by MNA (16%) and nicotinuric acid (NUA) (11.6%). The mean half-life for 2PY (calculated in urine) was 12.6 h [16].

MNA [17] and 2PY (molecular weight: 152.15 Da) are secreted by the kidney, though NAM itself is reabsorbed by renal tubules. This is why only small amounts of unmodified NAM appear in the urine—even after the administration of pharmacologically elevated doses of the compound. MNA (which presents a similar structure to 2PY) is an endogenous substrate of human organic cation transporter (hOCT2/SLC22A2) and members of the human (h) multidrug and toxins extrusion (MATE) 1 and hMATE2-K in renal tubules [18]. These ion transporters mediate renal excretion of both endogenous and exogenous substances. However, we should stress that there is yet no data available regarding the specific mechanisms of 2PY renal filtration/secretion. Plasma 2PY concentrations were first observed by Abelson, who calculated that the normal plasma concentrations in a fasting human are 200–600 ng/mL (1.3–4 µmol/L) [19]. Human plasma concentrations of 2PY increase with age [20]. Indeed, the mean plasma 2PY concentration in older healthy subjects (aged 50 to 90) was approximately 2.6-fold higher than the mean ± standard deviation (SD) value of 59 ± 33 ng/mL (0.39 ± 0.22 µmol/L) determined in young healthy subjects (aged five to 16). There was no gender difference in the plasma 2PY concentration. Age must therefore be taken into account when interpreting the results. Nutrition also influences metabolite concentrations. Shibata et al. investigated the metabolic interaction between nicotinic acid and NAM in rats fed large amounts of both compounds [21]. Under physiological conditions, niacin is chiefly catabolized to MNA, then to 4PY. However, NUA was the major catabolite when rats were fed a diet containing large amounts of nicotinic acid, and MNA was the major catabolite when the diet contained large amounts of NAM. When rats were fed a diet containing large amounts of both NUA and NAM, MNA and NUA were the major catabolites. Shibata et al. concluded that excess nicotinic acid and NAM are independently metabolized in rats.

## 3. Elevated 2PY Concentrations in Patients with Chronic Kidney Disease (CKD)

In one study, 2PY was isolated from the hemodialysis fluid of uremic patients (who were not consuming nicotinamic acid derivatives) [22], and it was suggested that the accumulation of 2PY in kidney failure might contribute directly to the signs and symptoms of uremia.

In a study of uremic rats, Rutkowski et al. have shown that NAM is present in the plasma, erythrocytes, lungs, liver, heart and brain, but is rarely detectable in fat tissue. NAM end-products accumulated in the liver, lungs and skeletal muscles, but not in fatty tissue or in the brain [23].

Carrey et al. measured erythrocyte levels of 2-pyridone-5-carboxamide ribonucleoside triphosphate (2PYTP) in uremic patients (molecular weight: 510.18 Da). The mean 2PYTP concentration was 11,122 ng/mL (21.8 µmol/L) in mild CKD but rose to 28,111 ng/mL (55.1 µmol/L) in patients with ESRD, to 36,172 ng/mL (70.9 µmol/L) in hemodialysis patients and even to 110,556 ng/mL (216.7 µmol/L) in patients undergoing continuous ambulatory peritoneal dialysis patients [24]. The plasma 2PY concentration was correlated with the erythrocyte 2PYTP concentration. Another study found that the mean ± SD plasma 2PY concentration is 126 ± 27 ng/mL (0.83 ± 0.18 µmol/L) in healthy subjects and 6086 ng/mL (40 µmol/L) in dialysis patients [25]. In the EUTox review of normal and pathologic concentrations of uremic toxins, Duranton et al. cited the following 2PY concentrations: the normal concentration in healthy subjects was 1370 ± 680 ng/mL (equivalent to 9.01 ± 4.47 µmol/L), which is higher than previously reported. The mean concentration in uremic patients was 4020 ± 3280 ng/mL (equivalent to 26.42 ± 21.56 µmol/L) and the highest concentration observed in uremic patients was 7800 ± 3590 ng/mL (equivalent to 51.26 ± 23.60 µmol/L) [26].

**Figure 2:**
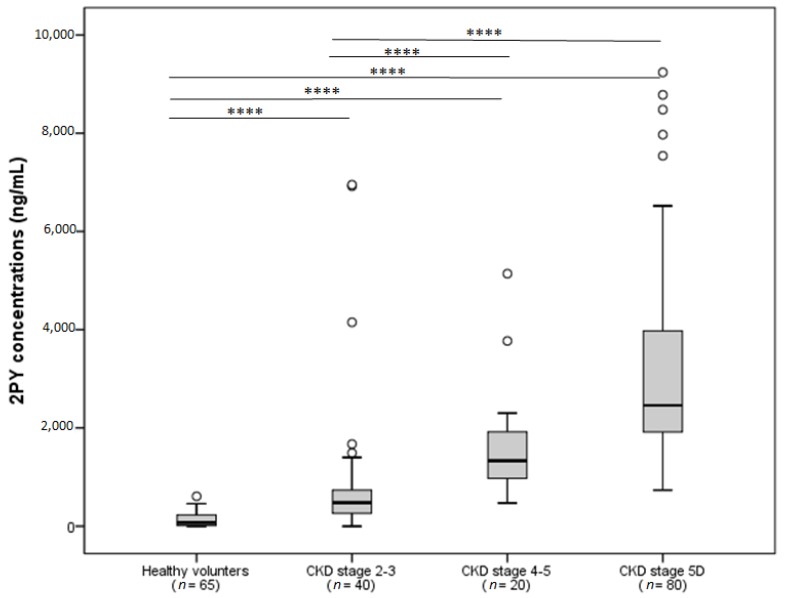
The 2PY concentration as a function of the chronic kidney disease (CKD) stage. To get the results in µmol/L, divide by the molecular weight (152.15 Da) **** *p* < 0.0001.

We recently used a sensitive, specific LC-MS/MS method to evaluate 2PY concentrations in patients at different stages of CKD (including 60 pre-hemodialysis patients and 80 hemodialysis patients) and in healthy volunteers (*n* = 65) (Figure 2). The advantages of the present method include a short run time, a simple sample preparation procedure (blood sample was deprotonated by acetonitrile), and a lower limit of quantification (50 ng/mL, equivalent to 0.32 µmol/L) compared to other techniques [10,11]. Our measurements confirmed the progressive rise in 2PY concentrations with successive CKD stages; the highest concentration was observed in hemodialysis patients (3100 ± 1870 ng/mL, equivalent to 20.37 ± 12.29 µmol/L) and greatly exceeded the value determined for healthy subjects (140 ± 20 ng/mL, equivalent to 0.92 ± 0.13 µmol/L) (Figure 2). The 2PY concentrations found in our 80 hemodialysis patients were comparable to those determined by the EUTox working group [26]. The mean age of our patients with CKD was 66 ± 13.

Only one study has assessed the elimination of 2PY during dialysis sessions. Due to its low molecular mass, 2PY was cleared by dialysis procedures. However, the 2PY concentration soon returned to its initial concentration after the dialysis session [24]. Successful kidney transplantation is the only way of permanently normalizing the 2PY serum concentration [24].

**Figure 3:**
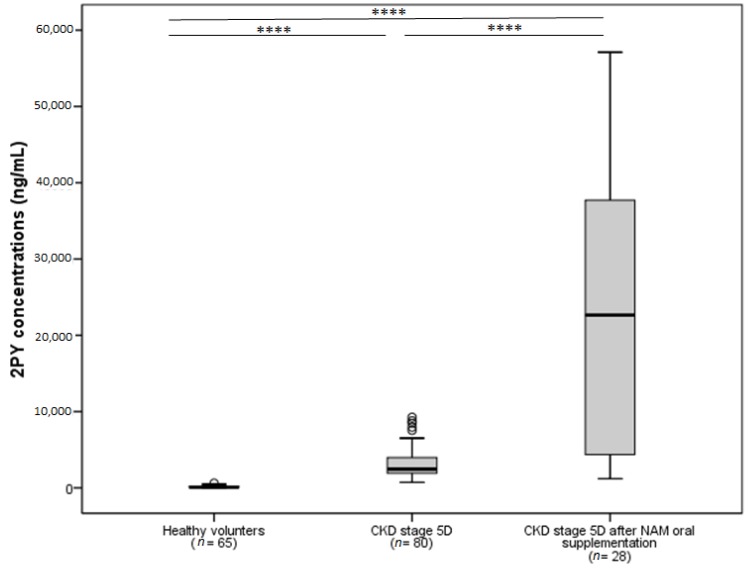
Comparison of 2PY concentrations in chronic kidney disease (CKD) patients stage 5D at baseline and after 24 weeks of oral supplementation with nicotinamide (NAM). To get the results in µmol/L, divide by the molecular weight (152.15 Da) **** *p* < 0.0001 (adapted from the NICOREN study [7]).

Accumulation of 2PY might be a problem when patients with CKD are supplemented with NAM. Consequently, we recently evaluated 2PY levels in NAM-supplemented hemodialysis patients as part of a non-inferiority study in which the effects of NAM and sevelamer on hyperphosphatemia in hemodialysis patients were compared (the NICOREN study) [7]. After 24 weeks of NAM treatment (mean dose in the maintenance phase: 1.3 g per day), 2PY levels in the NAM group were more than five times higher than at baseline (21,290 ± 17,750 ng/mL (139.93 ± 116.66 µmol/L) versus 3100 ± 1870 ng/mL (20.37 ± 12.29 µmol/L), *p* < 0.001) (Figure 3). Additional studies of 2PY levels in pre-dialysis CKD patients not receiving NAM supplements are currently required. This is an important issue, given that a randomized, placebo, controlled trial comparing nicotinamide (1500 mg daily), lanthanum carbonate (1000 mg three times daily), combined therapy or a dual placebo in 200 patients with a baseline eGFR of 20–45 mL/min/1.73 m² is currently underway [27].

## 4. Toxicological Profile

Rutkowski et al. were the first to describe 2PY as a uremic toxin [28]. Although the accumulation of 2PY in CKD patients might have toxic effects, the clinical consequences remain inconclusive. Some data suggest that an accumulation of 2PY could be harmful because the compound is known to inhibit the activity of poly(ADP-ribose) polymerase-1 (PARP-1) [28,29,30]. PARP-1 (the most active poly (ADP-ribose) polymerase) is an abundant nuclear enzyme that appears to be involved in the cellular response to DNA injury, since the latter triggers an increase in levels of this enzyme. Inhibition of poly-ADP-ribose formation tends to cause genomic instability and tumorigenesis in chronic models of DNA damage. The increased incidence of lymphopenia and cancer in CKD patients may be due (at least in part) to (i) lengthy, continuous exposure of the cell’s genome to endogenous DNA-damaging compounds and (ii) hypersensitivity to DNA-damaging agents in general as a result of PARP-1 inhibition. In experiments using a rat model, Synesiou et al. found that 4PY accumulation in erythrocytes during kidney failure is a marker for the accumulation of a related toxic NAD+ analogue that inhibits inosine monophosphate dehydrogenase in other cells [31]. Hence, the intra-erythrocyte metabolism of 4PY seems to influence renal anemia—at least in rats.

In contrast, the inhibition of PARP-1 can prevent several acute disease processes, including stroke, myocardial infarction and septic shock. In models of acute stress, PARP-1 inhibition may protect cellular NAD pools and prevent nuclear-factor-KB–dependent inflammatory signaling. Long-term protective roles of PARP-1 include DNA repair and the regulation of chromatin structure. Hence, 2PY’s ability to inhibit PARP-1 throughout the organism is a controversial topic. Although inhibition of PARP-1 has cytoprotective functions, excessive activation of the enzyme might lead to acute cell death. More importantly, NAM catabolites may inhibit several enzymes that are crucial for cell division and proliferation. High-dose nicotinamide is also used to treat patients outside the context of kidney failure. Chen et al. reported the results of a trial in 386 patients at high risk of skin cancer; the participants were randomized to nicotinamide 500 mg or a placebo twice daily for five years [32]. The researchers did not report on renal function but one can presume that it was normal. Participants randomized to nicotinamide were found to have a significantly lower risk of skin cancer than participants in the placebo group, and the two study groups did not have significantly different safety profiles.

Slominska et al. stated that the inhibition of PARP-1 activity could be beneficial, since it might prevent oxidative stress injury to endothelial cells by preserving the intracellular NAD pool and inhibiting apoptosis. Short-term exposure to elevated 2PY levels may therefore exert protective effects, whereas prolonged exposure is probably harmful (due to the impairment of DNA repair) [29].

**Figure 4:**
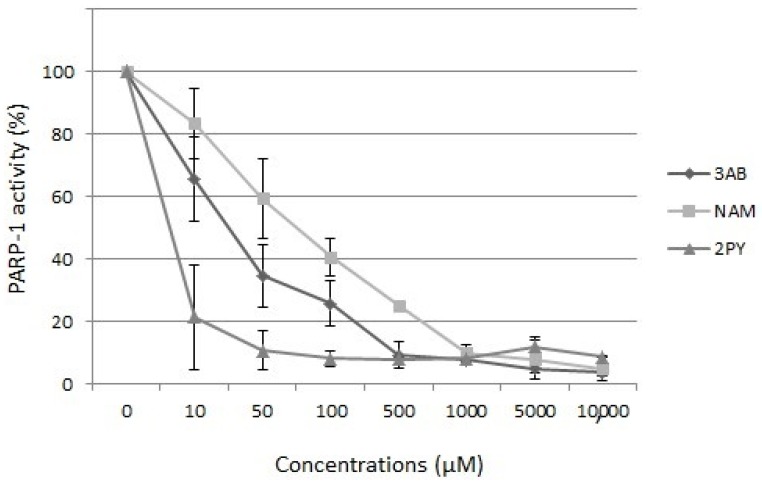
Effects of *N*-methyl-2-pyridone-5-carboxamide (2PY), nicotinamide (NAM) and 3-aminobenzamide (3AB) on the activity of purified poly (ADP-ribose) polymerase-1. Data are quoted as the mean ± SD (*n* = 6).

We recently studied 2PY’s effect on PARP-1 activity by using a colorimetric assay (the PARP Universal Colorimetric Assay Kit from R&D Systems). This assay provides information on whether (i) PARP is inhibited, (ii) DNA is damaged and (iii) the damaged DNA comes from non-apoptotic cells. Further, 3-aminobenzamide (a well-known PARP inhibitor) was used as a positive control. We found that 2PY and NAM inhibited PARP-1 activity in vitro, with IC50 values of 8 µM (1.22 mg/L) and 80 µM (12.17 mg/L), respectively (Figure 4)—confirming the report by Slominska et al. [29]. These IC50 values fall within the range of blood 2PY concentrations observed in dialysis patients.

In ESRD patients, thrombocytopenia constitutes another potential toxic effect of NAM. The first case report described the development of leukopenia and thrombocytopenia in a patient treated with niacin [33]. Both conditions resolved upon discontinuation. Rottembourg et al. [34] reported that six dialysis patients being treated with NAM 1 g/day developed marked thrombocytopenia within three months of treatment initiation. These results were confirmed by the NICOREN study [7]; four cases of thrombocytopenia were observed, and the platelet count fell to below 70,000/mm^3^ after four to eight weeks of treatment with NAM 1 g/day. It has since been reported that this adverse effect may result from a NAM-induced drop in the serum level of thyroxin-binding globulin or one of its derivatives. However, a putative causal relationship between 2PY and the genesis of thrombocytopenia in CKD patients has not yet been explored. Indeed, the PARP inhibitors commonly used to induce cell apoptosis in several types of cancer frequently induce hematologic disorders (and particularly thrombocytopenia) in a dose-dependent manner [35,36,37]. It remains to be determined whether the thrombocytopenia observed in our NAM-treated patients was related to PARP inhibition by accumulated 2PY.

Finally, several uremic toxins (i.e., indoxyl sulfate, para-cresyl sulfate) have been identified as likely to increase mortality and cardiovascular events in CKD [38,39,40]. Moreover, uremic toxins lead to tubular cell damage [41], inflammation and fibrosis [42,43]. It is noteworthy that high levels of 2PY in CKD patients affecting kidney function remains a possible issue, knowing 2PY’s actions on inflammation and DNA reparation, although these actions need to be evaluated. To explore this possibility, we followed a cohort of 60 pre-dialysis patients who had a dosage of circulating 2PY up to 2000 days. Among them, seven patients started hemodialysis during the follow-up. In univariate analysis, patients with the highest 2PY levels have a significantly high risk of initiating dialysis (*p* = 0.004). However, this predicting effect was not confirmed after adjustment on age or on CKD stages. Due to the small size of the cohort and the low number of events, further large studies are necessary to explore this issue.

## 5. Conclusions

The uremic toxin 2PY is back under the spotlight following the recent observation of its accumulation in patients supplemented with the phosphate binder NAM. The accumulation of 2PY may lead to several uremic toxic effects such as cancer and thrombocytopenia, although further research is needed to clarify this issue.
